# Auxiliary energy-assisted biodiesel production data from solid food waste oil

**DOI:** 10.1016/j.dib.2020.105456

**Published:** 2020-03-19

**Authors:** Miguel Carmona-Cabello, Javier Saez-Bastante, Sara Pinzi, M. Pilar Dorado

**Affiliations:** Department of Physical Chemistry and Applied Thermodynamics, EPS, Edificio Leonardo da Vinci, Campus de Rabanales, Universidad de Córdoba, Campus de Excelencia Internacional Agroalimentario ceiA3, 14071 Córdoba, Spain

**Keywords:** Restaurant residues, Ultrasound-assisted biodiesel, Food waste recycling, Biorefinery, Principal component analysis

## Abstract

A number of samples from solid food waste oil (SFWO) from different restaurants have been collected. Data regarding fatty acid profile, acid value, water content and kinematic viscosity were used for characterization purposes. Response surface methodology data has been used to carry out conventional transesterification optimization. The quality of the final product has been checked following the European biodiesel standard EN14214. To compare conventional and ultrasound-assisted transesterification results, energy consumption and reaction time data have been gathered. More information and result interpretation may be found in “Optimization of solid food waste oil biodiesel by ultrasound assisted transesterification” [1].

Specifications table

**Table:** 

Subject	Renewable Energy, Sustainability and the Environment
Specific subject area	Solid food waste recycling to produce biodiesel through ultrasound-assisted low-cost transesterification
Type of data	Tables Figures Excel file
How data were acquired	Gas chromatography, analytical analysis, Box–Behnken design, response surface methodology, mass spectrometry. Instruments: Perkin Elmer GC model Clarus 500, Rancimat Metrohm, Alcor CRT-160 by PAC, IKA bomb calorimeter, capillary-type viscometer Cannon-Fenske size 150, Karl Fischer titrator model DL32 Mettler Toledo, Seta Flash series 3 plus, HCO 342 Herzog by PAC, Statgraphics Centurion XVI software, QSonica LLC, Fluke power analyzers models 435 and 43B, Perkin Elmer mass spectrometer ICP-MS NexION 350X
Data format	Raw and analyzed
Parameters for data collection	Restaurants showing different customer habits and tastes (grill, fine dining, campus cafeteria and Italian restaurant) were selected. Seasonal implications were also considered. Only organic fraction was used for subsequent analysis.
Description of data collection	A set of 30 solid food waste oil samples were collected from four local restaurants. Sampling was conducted on random days during four months. Samples were homogenized and inorganic residues were discarded. Subsequently, organic fraction was milled, lyophilized for three days and stored at 4 °C.
Data source location	City/Town/Region: Cordoba Country: Spain
Data accessibility	With the article
Related research article	M. Carmona-Cabello, J. Sáez-Bastante, S. Pinzi, M.P. Dorado, Optimization of solid food waste oil biodiesel by ultrasound-assisted transesterification, Fuel, https://doi.org/10.1016/j.fuel.2019.115817

## Value of the Data

• These data provide physico-chemical and energy properties of a variety of restaurant organic residues that may be used to provide a recycling model through the concept of a biorefinery.
• Scientists working in biorefinery design and development may benefit from these data, besides biodiesel manufacturers.
• These data may be part of a wider pool of data, including agrifood residues, that may be used to design a valorization strategy.

## 1 Data description

In the excel file SFWO brief.xlsx, sheet no. 1, raw data related to characterization of solid food waste oil (SFWO), belonging to solid residues from tested restaurants, is provided [1]. Information shows fatty acid content and distribution, besides length of chain (LC) and total unsaturation degree (TU). Characterization also includes raw data of some of the most relevant physico-chemical properties (considering the feasibility of the conversion of this oil into biodiesel), namely acid value, water content and kinematic viscosity (Table 1).

**Table 1:** Sample physical and chemical properties. SD: standard deviation.

	Acid value, AV	Water content	Kinematic viscosity
	mgKOH/g	ppm	mm^2^/s
Sample 1	7.59	500	24.60
Sample 2	7.47	479	24.59
Sample3	7.54	584	24.61
Average	7.53	521	24.60
SD	0.06	65	0.01

For classification purposes, the comparison between a wide variety of oils and SFWO is provided by principal component analysis, shown in Table 2. Principal component 1 (PC1) includes oils with a combination of C16:0 and C18:1 fatty acids, while PC2 includes only the presence of C18:2.

**Table 2:** Principal component analysis. PC1: combination of C16:0 and C18:1; PC2: C18:2.

RAW MATERIALS		PC1	PC2
Common name	Binomial nomenclature	C16:0 & C18:1	C18:2
Solid food waste oil (SFWO)	*–*	−0.01030	0.38063
Yellow grease	*–*	−0.84551	0.31902
Brown grease	*–*	−0.58101	0.25712
Sunflower oil	*Helianthus annuus* oil	2.15595	−0.40731
Rice bran oil	*Oryza sativa* bran oil	0.74175	0.37730
Corn oil	*Zea mays* oil	1.34799	0.05043
Rapeseed oil	*Brassica napus* oil	0.09923	0.75998
Crambe oil	*Crambe cordifolia* and *C. abyssinica* oils	−0.44872	−0.58297
Canola oil	*Brassica rapa, B. juncea* and *B. napus* oil	−0.10663	0.90778
Sesame oil	*Sesamum indicum* oil	0.52511	0.55850
Peanut oil	*Arachis hypogaea* oil	0.32027	0.58830
Coconut oil	*Cocos nucifera* oil	−1.11843	−1.38740
Olive oil	*Olea europaea* oil	−0.75998	1.41586
Jatropha oil	*Jatropha curcas* oil	0.68927	0.15252
Almond oil	*Prunus dulcis* oil	1.25989	0.06333
Castor oil	*Ricinus communis* oil	−2.71850	−2.83953
Lineseed oil	*Linum usitatissimum* oil	−0.10601	−0.99837
Walnut oil	*Juglans regia* oil	1.92387	−0.74607
Walnut kernel oil		1.76091	−0.71735
Poppyseed oil	*Papaver somniferum* oil	2.29905	−0.59814
Soybean oil	*Glicine* max oil	1.30947	−0.71286
Cotton oil	*Gossypium hirsutum* oil	1.70090	−0.65943
Groundnut oil	*Arachis villosulicarpa* oil	0.28692	0.48147
Hazelnut oil	*Corylus avellane* oil	−0.16128	1.93525
*Neem oil*	*Azadirachta indica* oil	−0.71399	0.56460
Karanja oil	*Millettia pinnata* oil	−0.77982	0.75303
Mustard	*Sinapis alba* oil	−0.39443	−1.23350
Abyssiniam mustard	*Brassica carinata* oil	−0.17655	−1.28249

Transesterification was preceded by acid esterification, due to the high oil acid value. Raw data about evolution and reduction of the acid value during esterification is shown in Table 3.

**Table 3:** Evolution of acid value during acid esterification (pre-treatment before transesterification) of solid food waste oil (SFWO).

Acid value (mg KOH/mg)	Free fatty acid content (% w/w)
7.53	3.765
2.19	1.095
1.78	0.890
1.24	0.620
0.61	0.305
0.38	0.190
0.31	0.155
0.28	0.140

Sheet no. 2 (excel file SFWO brief.xlsx) shows gas chromatography results (raw and analysed data) from the analysis carried out following a design of experiments (DOE) for SFWO transesterification. Fatty acid content was provided, besides ester yield, before and after cleaning process. Table 4 includes resulting fatty acid methyl ester (FAME) yield (measured by gas chromatography) under both conventional transesterification (CT) and ultrasound-assisted transesterification (UT), including standard deviation (SD).

**Table 4:** Fatty acid methyl ester (FAME) yield under conventional transesterification (CT) and ultrasonication conditions (UT); IS: internal standard; SD: standard deviation.

CT, test 1					
Time (s)	IS weight (mg)	IS area	Sample weight (mg)	Sum area	Yield (%)
30	49.685	131,795.50	305.56	732,246.72	74.08
60	49.685	131,559.48	233.07	653,112.66	84.51
120	50.389	94,232.13	242.50	494,636.43	88.29
300	50.389	93,198.96	233.20	474,155.35	88.32
600	50.394	137,183.22	236.89	712,625.21	89.23
1200	49.685	91,297.18	243.00	499,055.05	91.32
1500	49.685	91,297.18	242.30	499,055.05	91.58
1800	50.389	95,049.29	244.20	518,259.41	91.88
2400	50.289	91,297.18	242.30	499,055.05	92.70
3600	50.289	92,646.79	233.50	497,752.07	94.17

CT, test 2					
Time (s)	IS weight (mg)	IS area	Sample weight (mg)	Sum area	Yield (%)
30	49.685	131,795.50	305.69	732,246.72	74.05
60	49.685	131,559.48	233.10	653,112.66	84.50
120	50.389	94,232.13	242.60	494,636.43	88.26
300	50.389	95,049.43	243.00	501,924.73	88.76
600	50.394	137,183.22	236.94	712,625.21	89.22
1200	49.685	91,297.18	243.10	499,055.05	91.28
1500	49.685	91,297.18	242.15	499,055.05	91.64
2400	50.389	95,049.29	244.20	518,259.41	91.87
1800	50.289	91,297.18	242.30	499,055.05	92.70
3600	50.289	92,646.79	233.45	497,752.07	94.19

UT, test 1					
Time (s)	IS weight (mg)	IS area	Sample weight (mg)	Sum area	Yield (%)
5	50.389	131,795.50	305.69	801,246.72	83.73
10	50.389	131,559.48	222.70	620,012.66	84.01
30	50.389	97,932.13	241.20	494,636.43	84.63
60	50.389	96,932.13	241.20	494,636.43	85.71
120	50.389	96,547.70	221.00	466,988.05	87.48
300	50.389	91,015.18	254.80	499,055.05	88.66
600	49.685	91,097.18	249.30	499,055.05	89.25
1200	50.389	95,030.29	242.02	518,259.41	92.73
1500	50.289	91,297.18	242.40	499,055.05	92.66
1800	50.289	91,490.79	241.00	497,752.07	92.66
2400	49.685	91,490.79	238.00	497,752.07	92.70
3600	50.289	91,297.18	242.40	499,055.05	92.66

UT, test 2					
Time (s)	IS weight (mg)	IS area	Sample weight (mg)	Sum area	Yield (%)
5	50.389	133,795.50	300.90	801,246.72	83.54
10	50.389	130,959.48	223.90	620,012.66	84.04
30	50.389	97,932.13	241.20	494,636.43	84.63
60	50.389	96,932.13	241.20	494,636.43	85.71
120	50.389	96,647.70	220.30	466,988.05	87.65
300	50.389	91,015.18	254.80	499,055.05	88.66
600	50.389	91,097.18	249.30	499,055.05	90.52
1200	50.389	94,930.29	242.02	518,259.41	92.84
1500	50.389	91,297.18	243.40	499,055.05	92.46
1800	50.389	91,570.79	240.00	497,752.07	92.84
2400	50.389	91,297.18	242.40	499,055.05	93.13
3600	50.389	91,284.79	239.00	497,752.07	93.88

Conventional transesterification (CT) (average between tests 1 & 2)					
Time	Test 1	Test 2	Average	SD	
s	Yield (%)				
30	74.08	74.05	74.07	0.02	
60	84.51	84.50	84.51	0.01	
120	88.29	88.26	88.28	0.03	
300	88.32	88.76	88.54	0.31	
600	89.23	89.22	89.23	0.01	
1200	91.32	91.28	91.30	0.03	
1500	91.58	91.64	91.61	0.04	
1800	91.88	91.87	91.87	0.01	
2400	92.70	92.66	92.68	0.03	
3600	94.17	94.19	94.18	0.01	

Ultrasound assisted-transesterification (UT) (average between tests 1 & 2)					
Time	Test 1	Test 2	Average	SD	
s	Yield (%)				
5	83.73	83.54	83.64	0.13	
10	84.01	84.04	84.03	0.03	
30	84.63	84.63	84.63	0.00	
60	85.71	85.71	85.71	0.00	
120	87.48	87.65	87.57	0.12	
300	88.66	88.66	88.66	0.00	
600	89.25	90.52	89.89	0.90	
1200	92.73	92.84	92.79	0.08	
1500	92.66	92.46	92.56	0.14	
1800	92.66	92.84	92.75	0.13	
2400	92.70	93.13	92.91	0.30	
3600	93.20	93.88	93.54	0.48	

**Fig. 1:**
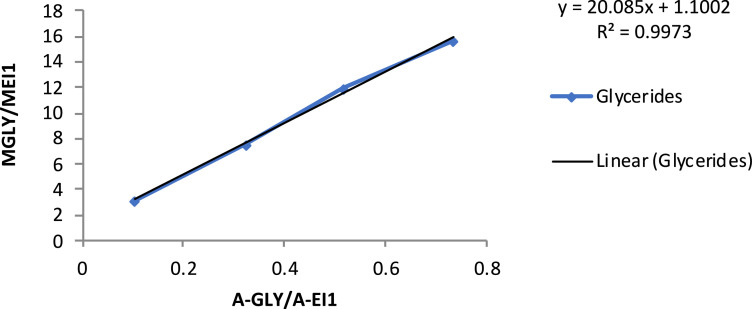
Glyceride content calibration curve. MGLY: glyceride concentration; MEI1: internal standard concentration; A-GLY: glyceride area; A-EI1: internal standard area.

**Fig. 2:**
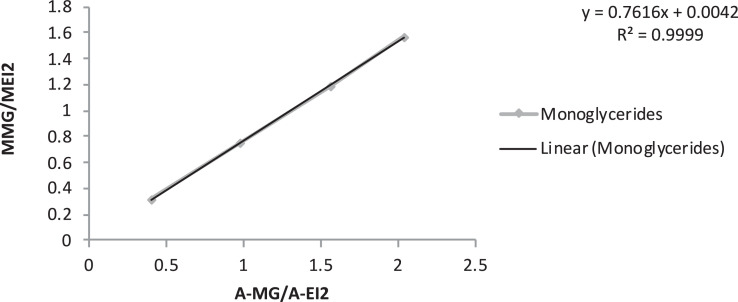
Monoglyceride content calibration curve. MMG: monoglyceride concentration; MEI2: internal standardconcentration; A-MG: monoglyceride area; A-EI2: internal standard area.

**Fig. 3:**
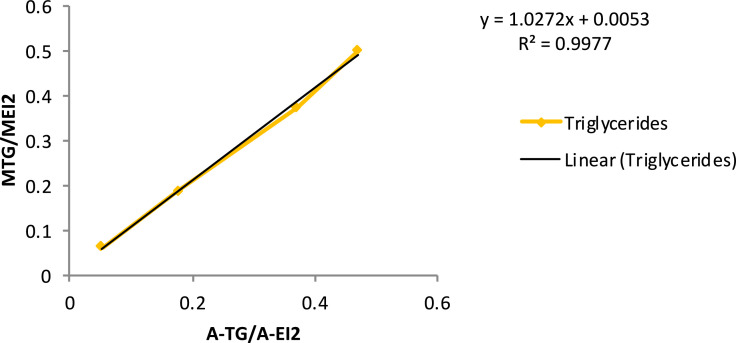
Triglyceride content calibration curve. MTG: triglyceride concentration; MEI2: internal standard concentration; A-TG: triglyceride area; A-EI2: internal standard area.

**Fig. 4:**
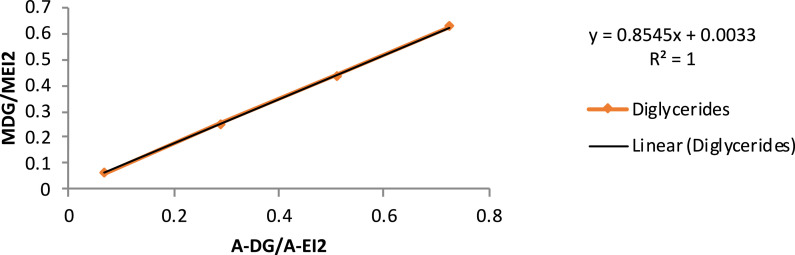
Diglyceride content calibration curve. MDG: diglyceride concentration; MEI2: internal standard concentration; A-MG: triglyceride area; A-AEI2: internal standard area.

Table 5 exhibits the trend of glyceride (mono-, di- and triglycerides) concentration vs*.* time, during ultrasound-assisted transesterification. Calibration curves are also provided (Table 6 and Fig. 1, Fig. 2, Fig. 3, Fig. 4).

**Table 5:** Glyceride concentration vs. time during ultrasound assisted transesterification. Dly: glycerides, TG: triglycerides, DG: diglycerides, MG: monoglycerides.

Time (s)	sample (mg)	EI1 (mg)	EI2 (mg)	EI1 (surface)	EI2 (surface)	Gly (surface)	MG (surface)	DG (surface)	TG (surface)	Gly (%)	MG (%)	DG (%)	TG (%)
0											0.00	0.00	100.00
5	44.90	0.07	0.40	11,217.00	32,661.00	1522.00	86,288.00	33,293.00	72,415.80	0.56	1.79	0.78	2.63
10	46.80	0.07	0.40	9337.00	27,647.00	2235.00	54,503.00	20,671.00	39,325.00	0.83	1.30	0.55	1.27
30	53.90	0.07	0.40	10,538.00	34,745.00	2218.00	56,467.00	22,492.00	43,787.00	0.65	0.93	0.42	0.98
60	52.00	0.07	0.40	10,662.00	33,406.00	1422.00	48,474.00	20,940.00	41,353.00	0.48	0.86	0.42	0.99
120	53.10	0.07	0.40	11,547.00	33,760.00	391.00	40,239.00	12,299.00	39,575.00	0.22	0.69	0.24	0.92
300	53.11	0.07	0.40	12,159.00	29,074.00	2104.00	30,171.00	6409.00	26,646.00	0.57	0.60	0.15	0.72
600	55.17	0.07	0.40	11,717.00	33,258.00	783.00	34,630.00	3649.00	28,996.00	0.29	0.58	0.07	0.66
1800	53.69	0.07	0.40	11,670.00	32,624.00	374.00	24,500.00	3978.00	28,229.00	0.21	0.43	0.08	0.67

**Table 6:** Calibration curve data.

	EI1 (μg)	EI2	GLY	MG	DG	TG	MGLY/MEI1	MMG/MEI2	MDG/MEI2	MTG/MEI2	A-gly	A-EI1	A-MG
Solution 1	80	800	5.1	101.8	49.0	49.9	1.27	0.13	0.06	0.06	975.72	7891.96	9209.15
Solution 2	80	800	15.4	254.4	98.0	99.9	3.18	0.32	0.12	0.12	2160.83	8437.27	16,940.57
Solution 3	80	800	25.7	508.8	196.0	199.7	6.36	0.64	0.25	0.25	3309.88	7707.42	37,217.30
Solution 4	80	800	51.3	1018	490.1	499.3	12.72	1.27	0.61	0.62	6271.17	8430.81	73,333.05
	A-EI2	A-DG		A-TG	A-GLY/A-EI1	A-MG/A-EI2	A-DG/A-EI2	A-TG/A-EI2	MGLY/MEI1	MMG/MEI2			

Solution 1	38,075.73	2218.31	1799.46	1485.39	0.12	0.24	0.06	0.04	3.13	0.31			
Solution 2	39,582.00	5405.35	6019.57	3626.52	0.26	0.43	0.14	0.09	7.51	0.75			
Solution 3	43,923.84	11,869.14		10,731.97	0.43	0.85	0.27	0.24	11.88	1.19			
Solution 4	23,846.00	31,824.57		20,508.81	0.74	3.08	1.33	0.86	15.63	1.56			
	MDG/MEI2	MTG/MEI2	A-GLY/A-EI1	A-MG/A-EI2	A-DG/A-EI2	A-TG/A-EI2							

Solution 1	0.063	0.063	0.105	0.400	0.069	0.052							
Solution 2	0.250	0.188	0.324	0.982	0.288	0.178							
Solution 3	0.438	0.375	0.516	1.565	0.511	0.372							
Solution 4	0.624	0.501	0.735	2.036	0.725	0.473

Table 7 show energy analysis to compare energy consumption under both conditions, namely conventional and ultrasound-assisted transesterification. For this purpose, a new “energy use index” parameter has been defined (Eq. (1)).

(1) EUI = LHV /CE

**Table 7:** Energy use index (EUI) to compare conventional and ultrasound-assisted transesterification; SD: standard deviation.

Parameters	Esterification+ conventional transesterification	Esterification + ultrasound-assisted transesterification
FIRST STEP: ESTERIFICATION		
Low calorific vale (J/g)	37,032.24	37,032.24
Amount of consumed energy, previous esterification (J/g)	31,500	31,500
Mass unit sample 1 (g)	12.11	12.11
Mass unit sample 2 (g)	11.43	11.43
Mass unit sample 3 (g)	11.70	11.70
EUI 1	14.24	14.24
EUI 2	13.44	13.44
EUI 3	13.75	13.75
EUI average	13.81	13.81
SD	0.40	0.40
SECOND STEP: TRANSESTERIFICATION		
Low calorific vale (J/g)	37,032.24	37,032.24
Amount of consumed energy during transesterification 1 (J/g)	378,000	90,398
Amount of consumed energy during transesterification, repetition 2 (J/g)	n.d.	81,968
Amount of consumed energy during transesterification, repetition 3 (J/g)	n.d.	91,413
Mass unit sample 1 (g)	12.11	14.06
Mass unit sample 2 (g)	11.43	12.60
Mass unit sample 3 (g)	11.7	13.82
EUI 1	1.19	5.76
EUI 2	1.12	5.69
EUI 3	1.15	5.60
EUI average	1.15	5.68
SD	0.03	0.05
Consumed energy (EUI)	Average	SD
EUI previous esterification	13.81	0.33
EUI conventional transesterification	1.15	0.03
EUI ultrasound Transesterification	5.68	0.07

Where, LHV is low calorific value (J/g) and CE is the amount of energy per mass unit required for its synthesis (J/g). Table 8 includes biodiesel properties, following European biodiesel standard EN 14,214. Finally, Table 9 includes a detailed quantitative analysis of metal content by inductivity coupled plasma mass spectrometry (ICP-MS).

**Table 8:** Quality analysis of biodiesel from solid food waste oil following European standard EN 14214; CFPP: cold filter plugging point; Gly: glycerides; MD: monoglycerides; DG: diglycerides; TG: triglycerides; SD: standard deviation.

	EN 14214	Experimental data from conventional transesterification				
Quality parameters	Method and threshold	sample 1	Sample 2	Sample 3	Average	SD
Water content (mg/g)	EN ISO 12937; Max: 500	281.46	271.50	194.06	249.00	47.85
Kinematic viscosity at 40 °C (mm^2^/s)	EN ISO 3104; 3.5–5.0	4.03	4.10	4.17	4.10	0.07
Density at 15 °C (g/L)	EN ISO 3675; 860–900	870	871	869	870	1
CFPP ( °C)	EN 116	−4.0	−4.0	−4.0	−4.0	0.0
Low calorific value (J/g)	ASTM D240; Min: 35,000	39,339.00	39,530.00	39,493.00	39,454.00	103.18
Oxidation stability (h)	EN 14112; Min: 8	2.16	2.10	2.05	2.10	0.06
Flash point ( °C)	EN ISO 3679; Min: 101	165	167	166	166	1
Carbon residue (% w/w)	EN ISO 10,370; Max: 0.30	0.045	0.013	0.020	0.026	0.02
Acid value (mg KOH/g)	EN 14104; Max: 0.50	0.150	0.170	0.160	0.160	0.010

Quantitative analysis by inductivity coupled plasma mass spectrometry (ICP-MS) Conventional transesterification				
	Sample 1	Sample 2	Average	SD
Na (ppm)	5.015	5.192	5.100	0.130
K (ppm)	0.653	0.730	0.690	0.050
Mg (ppm)	0.099	0.064	0.082	0.024
Cu (ppb)	1233.224	1196.420	1214.000	26.710

	EN 14214	Experimental data of ultrasound-assisted transesterification				
Quality parameters	Method and threshold	sample 1	Sample 2	Sample 3	Average	SD
Water content (mg/g)	EN ISO 12937; Max: 500	280.60	472.25	387.16	380.00	96.02
Kinematic viscosity at 40 °C (mm^2^/s)	EN ISO 3104; 3.5–5.0	4.17	4.20	4.31	4.23	0.07
Density at 15 °C (g/L)	EN ISO 3675; 860–900	880	880	880	880	1
CFPP ( °C)	EN 116	−4.0	−3.0	−4.0	−3.7	0.6
Low calorific value (J/g)	ASTM D240; Min: 35,000	39,625.0	39,585.0	39,506.0	39,572.0	60.6
Oxidation stability (h)	EN 14112; Min: 8	3.18	3.22	3.35	3.25	0.08
Flash point ( °C)	EN ISO 3679; Min: 101	160	165	164	163	3
Carbon residue (% w/w)	EN ISO 10370; Max: 0.30	0.0458	0.0120	0.0205	0.0261	0.0176
Acid value (mg KOH/g)	EN 14104; Max: 0.50	0.17	0.16	0.17	0.17	0.01

Quantitative analysis by inductivity coupled plasma mass spectrometry (ICP-MS)				
Ultrasound-assisted transesterification				
	Sample 1	Sample 2	Average	SD
Na (ppm)	5.254	5.147	5.200	0.080
K (ppm)	0.63	0.71	0.67	0.05
Mg (ppm)	0.092	0.071	0.081	0.015
Cu (ppb)	1223.00	1187.00	1205.00	25.45

sample	sample (mg)	EI1 (mg)	EI2 (mg)	Area EI1	Area EI2	Area Gly	Area MG	Area DG	Area TG	Gly (%)	MG (%)	DG (%)	TG (%)
**Conventional transesterification**	54.98	0.07	0.40	11,208.00	28,116.00	3272.00	38,877.00	16,284.00	26,547.00	0.83	0.78	0.37	0.72
**Ultrasound-assisted transesterification**	54.30	0.07	0.40	11,901.00	29,821.00	2934.00	22,933.00	9984.00	23,263.00	0.73	0.44	0.22	0.60

**Table 9:** Detailed quantitative analysis of metal content by inductivity coupled plasma mass spectrometry (ICP-MS). Initial sample quantity (mg): 507.9; sample preparation volume (mL): 10.0; aliquot volume (mL): 1.0; diluted to volume (mL): 10.0.

Element	Mass (ppb)	Intensity
H	–	–
He	–	–
Li	0.000	0
Be	5.587	10
B	86.939	65
C	0.000	0
N	145,333,219.428	131,014
O	–	–
F	–	6454
Ne	0.000	0
Na	3774.583	26,439
Mg	0.000	0
Al	0.000	0
Si	0.000	0
P	0.000	0
S	8553.083	2875
Cs	0.000	0
Ar	0.000	0
K	0.000	0
Ca	0.000	0
*Sc*	0.000	0
Ti	0.000	0
V	0.000	0
Cr	0.000	0
Mn	0.000	0
Fe	0.000	0
Co	0.000	0
Ni	0.000	0
Cu	1181.982	81,545
Zn	1520.834	25,832
Ga	0.000	0
Ge	0.000	0
As	0.000	0
Se	0.000	0
Br	103.552	63
Kr	0.000	0
Rb	0.000	0
Sr	0.000	0
Y	0.000	0
Zr	0.000	0
Nb	0.000	0
Mo	0.000	0
Ru	0.000	0
Rh	0.000	0
Pd	0.000	0
Ag	0.000	0
Cd	0.000	0
In	0.000	0
Sn	0.000	0
Te	0.000	0
I	278.157	1591
Xe	0.000	0
Cs	0.000	0
Ba	0.000	0
La	0.130	17
Ce	0.000	0
Pr	0.000	0
Nd	0.000	0
Sm	0.000	0
Eu	0.057	10
Gd	0.000	0
Tb	0.346	74
Dy	0.000	0
Ho	0.337	76
Er	0.304	69
Tm	0.137	34
Yb	0.000	0
Lu	0.196	35
Hf	0.000	0
Ta	0.000	0
W	0.000	0
*Re*	0.000	0
Os	0.000	0
Ir	0.000	0
Pt	0.164	17
Au	0.000	0
Hg	0.000	0
Tl	0.311	65
Pb	63.352	12,939
Bi	0.449	71
Th	0.000	0
U	0.000	0

## 2 Experimental design, materials, and methods

After collecting SFW samples from four restaurants during several weeks and seasonally (see [1] for more details) and once inorganic residues were discarded (plastics, etc.) they were mixed together, homogenized, lyophilized and stored at 4 °C, oil was extracted using Soxhlet method. Lipids were winterized under centrifugation at 2000 rpm, during 10 min, at 0 °C, as explained in [1]. For each analysis, three replicates were considered (samples 1–3), while four points were used to design each calibration curve. Oil was characterized as previously mentioned. Principal component analysis was used to classify the lipids considering most frequently used oils to provide biodiesel through transesterification. Acid value was measured to check whether a pre-treatment consisting in an acid esterification, prior to transesterification, was needed. Experimental design was performed with Statgraphics Centurion XVI software and Box-Behnken design [1].

Ultrasound-assisted transesterification was carried out with a sonicator probe Q700 QSonica LLC, under a frequency of 20 kHz, 100% duty cycle and 50% amplitude. The consumption of energy was analyzed using Eq. (1) and two Fluke power analyzers working at 1000 V rms and 1250 V rms, respectively. More details are provided in reference [1]. Biodiesel characterization was carried out following European biodiesel standard EN 14,214. Metal content was analyzed using by ICP-MS.

## Declaration of Competing Interest

The authors declare that they have no known competing financial interests or personal relationships which have, or could be perceived to have, influenced the work reported in this article.

## Appendix Supplementary materials

Image, application 1
